# 18F-FDG primary tumor uptake to improve N status prediction in cT1 non-metastatic non-small cell lung cancer: development and validation of a positron emission tomography model

**DOI:** 10.3389/fmed.2023.1141636

**Published:** 2023-04-26

**Authors:** David Morland, Marco Chiappetta, Pierre-Emmanuel Falcoz, Marie-Pierre Chenard, Salvatore Annunziata, Luca Boldrini, Filippo Lococo, Alessio Imperiale

**Affiliations:** ^1^Médecine Nucléaire, Institut Godinot, Reims, France; ^2^CReSTIC EA 3804 et Laboratoire de Biophysique, Université de Reims Champagne-Ardenne, Reims, France; ^3^Unità di Medicina Nucleare, GSTeP Radiofarmacia, TracerGLab, Dipartimento di Radiologia, Radioterapia ed Ematologia, Fondazione Policlinico Universitario A. Gemelli IRCCS, Rome, Italy; ^4^Università Cattolica del Sacro Cuore, Rome, Italy; ^5^Chirurgia Toracica, Fondazione Policlinico Universitario A. Gemelli IRCCS, Rome, Italy; ^6^Service de Chirurgie Thoracique, Hôpitaux Universitaires de Strasbourg, Strasbourg, France; ^7^Service de Pathologie, Hôpitaux Universitaires de Strasbourg, Strasbourg, France; ^8^Unità di Radioterapia, Radiomics, Dipartimento di Radiologia, Radioterapia ed Ematologia, Fondazione Policlinico Universitario A. Gemelli IRCCS, Rome, Italy; ^9^Médecine Nucléaire, Institut de Cancérologie Strasbourg Europe (ICANS), Strasbourg, France; ^10^Hôpitaux Universitaires de Strasbourg, Faculté de Médecine, Université de Strasbourg, Strasbourg, France; ^11^DRHIM, IPHC, UMR7178, CNRS/Unistra, Strasbourg, France

**Keywords:** NSCLC, lymph nodes, positron emission tomography, FDG, model

## Abstract

**Purpose:**

Occult lymph node involvement is a major issue in the management of non-small cell lung carcinoma (NSCLC), with an estimated prevalence of approximately 2.9–21.6% in 18F-FDG PET/CT series. The aim of the study is to construct a PET model to improve lymph node assessment.

**Methods:**

Patients with a non-metastatic cT1 NSCLC were retrospectively included from two centers, one used to constitute the training set, the other for the validation set. The best multivariate model based on Akaike’s information criterion was selected, considering age, sex, visual assessment of lymph node (cN0 status), lymph node SUVmax, primary tumor location, tumor size, and tumoral SUVmax (T_SUVmax). A threshold minimizing false pN0 prediction was chosen. This model was then applied to the validation set.

**Results:**

In total, 162 patients were included (training set: 44, validation set: 118). A model combining cN0 status and T_SUVmax was selected (AUC 0.907, specificity at threshold: 88.2%). In the validation cohort, this model resulted in an AUC of 0.832 and a specificity of 92.3% versus 65.4% for visual interpretation alone (*p* = 0.02). A total of two false N0 predictions were noted (1 pN1 and 1 pN2).

**Conclusion:**

Primary tumor SUVmax improves N status prediction and could allow a better selection of patients who are candidates for minimally invasive approaches.

## 1. Introduction

The prediction of lymph node involvement (LNI) is a major challenge in the management of non-small cell lung tumors (NSCLC), as illustrated in the eighth edition of the TNM classification (1). LNI is strongly correlated with overall survival and disease-free interval (2, 3). Regardless of T status, the 5-year survival rates decrease from 56% in pathologic (p) pN0 patients to 38% (pN1), 26% (pN2), and 6% in pN3 disease (4). 18F-Fluorodeoxyglucose (18F-FDG) positron emission tomography coupled with computed tomography (PET/CT) is considered the reference examination for staging NSCLC. However, the sensitivity of PET/CT for the prediction of LNI is insufficient to dispense with surgical and pathological confirmation. The prevalence of occult pN2 disease in patients with clinical stage I NSCLC is estimated to be 6.5% (5), ranging from 2.9 to 21.6% in patients with peripheral and central tumors, respectively (6, 7).

Improving LNI assessment would open interesting perspectives, especially in early-stage NSCLC. For resectable tumors, as thoracotomy is progressively supplanted by mini-invasive approaches, such as video-assisted thoracic surgery (8), it would help to solve the dilemma of lymphadenectomy extension (8) and secures the decision whether to perform postoperative chemotherapy. For unresectable tumors, it would allow better planning of radiotherapy and possible adjuvant treatments. Overall, some 18F-FDG PET/CT predictors of occult LNI have already been described: primary tumor localization as central and right superior lobe tumors are associated with a greater risk of occult N2 node (6); primary tumor size: the negative predictive value of 18F-FDG PET/CT was higher for tumors of less than 3 cm in diameter (9–11); primary tumor 18F-FDG uptake (11–13). The objective of this study is to build and validate a model to identify patients without LNI more accurately than the simple visual interpretation of 18F-FDG PET/CT. We will focus on patients suitable for mini-invasive surgery, namely T1 non-metastatic patients.

## 2. Materials and methods

### 2.1. Patients

Patients satisfying the following criteria were included in the study: histologically proven NSCLC; available baseline PET/CT data; clinical (c) T1 and clinical M0 status (maximum diameter of primary tumor of less than 3 cm, as measured on baseline PET/CT and no visible metastasis); available pathological results with pN status; delay between PET/CT and pathological results of less than 2 months. Exclusion criteria were as follows: benign lung disease; non-FDG-avid subtypes (lepidic adenocarcinoma, carcinoid tumors); neoadjuvant systemic therapy performed prior to PET/CT.

A total of two data sources were used as follows: a training database consisting of patients with lung cancer referred to Strasbourg University Hospital (Strasbourg, France) between July 2004 and September 2009 for preoperative PET/CT; a validation database consisting in patients referred to Policlinico Universitario A. Gemelli (Rome, Italy) between January 2018 and December 2021.

### 2.2. PET/CT acquisition and interpretation

After checking patients’ blood glucose levels (<2 g/l), staging PET/CT was performed at least from the skull base to the proximal thigh using two different PET/CT machines and acquisition protocols.

For the training cohort, images were acquired 60 min after an intravenous administration of 5 mg of diazepam, 80 mg of phloroglucinol, and 5.5 MBq/kg of 18F-FDG. A Discovery ST system (GE Medical System, Milwaukee, United States) was used. CT (140 kV, 80 mAs) was acquired first, followed by a two-dimensional PET/CT acquisition (seven fields of view of 15 cm, 4 min/field). PET data were reconstructed using an Ordered Subset Expectation Maximization algorithm (OSEM 2 iterations, 15 subsets, 128 × 128 matrix, slice thickness: 3.27 mm). PET/CT was reviewed on-site by two nuclear medicine physicians, with 4 and 14 years of experience in nuclear medicine, on a Xeleris workstation (GE Medical System, Milwaukee, United States).

For the validation cohort, images were acquired 60 min after the administration of 3 MBq/kg of 18F-FDG. A Biograph mCT (Siemens Healthineers) PET/CT was used, using the following parameters: CT (120 kV, 50 mAs, slice thickness: 3 mm), PET (2.5 min/position, reconstruction with OSEM algorithm: two iterations, 21 subsets, voxel size: 3.2 mm × 3.2 mm × 5 mm). Again, images were interpreted by two experienced nuclear medicine physicians (5 and 12 years of experience) on a SyngoVia workstation (Siemens Healthineers).

### 2.3. Surgical staging

In all cases, lymph node dissection systematically included at least three mediastinal stations including station 7, as recommended in the study of (14). In the training cohort, furthermore, systematic lymph node dissection was performed.

### 2.4. Data collection

The following parameters were collected as follows: sex, date of birth, date of diagnosis, histology of the primary tumor, and pN status. Regarding PET/CT studies, the following characteristics were measured on the training set: upper right lobe location of the primary tumor (yes/no); central location of the primary (yes/no); cT status derived from the diameter of the primary measured on the CT part of the PET/CT; cN status based on visual interpretation; 18F-FDG uptake (SUVmax) of both primary and nodal stations, denoted as T_SUVmax and N_SUVmax, respectively. Only features identified as relevant in the model were measured on the validation set.

**Figure 1:**
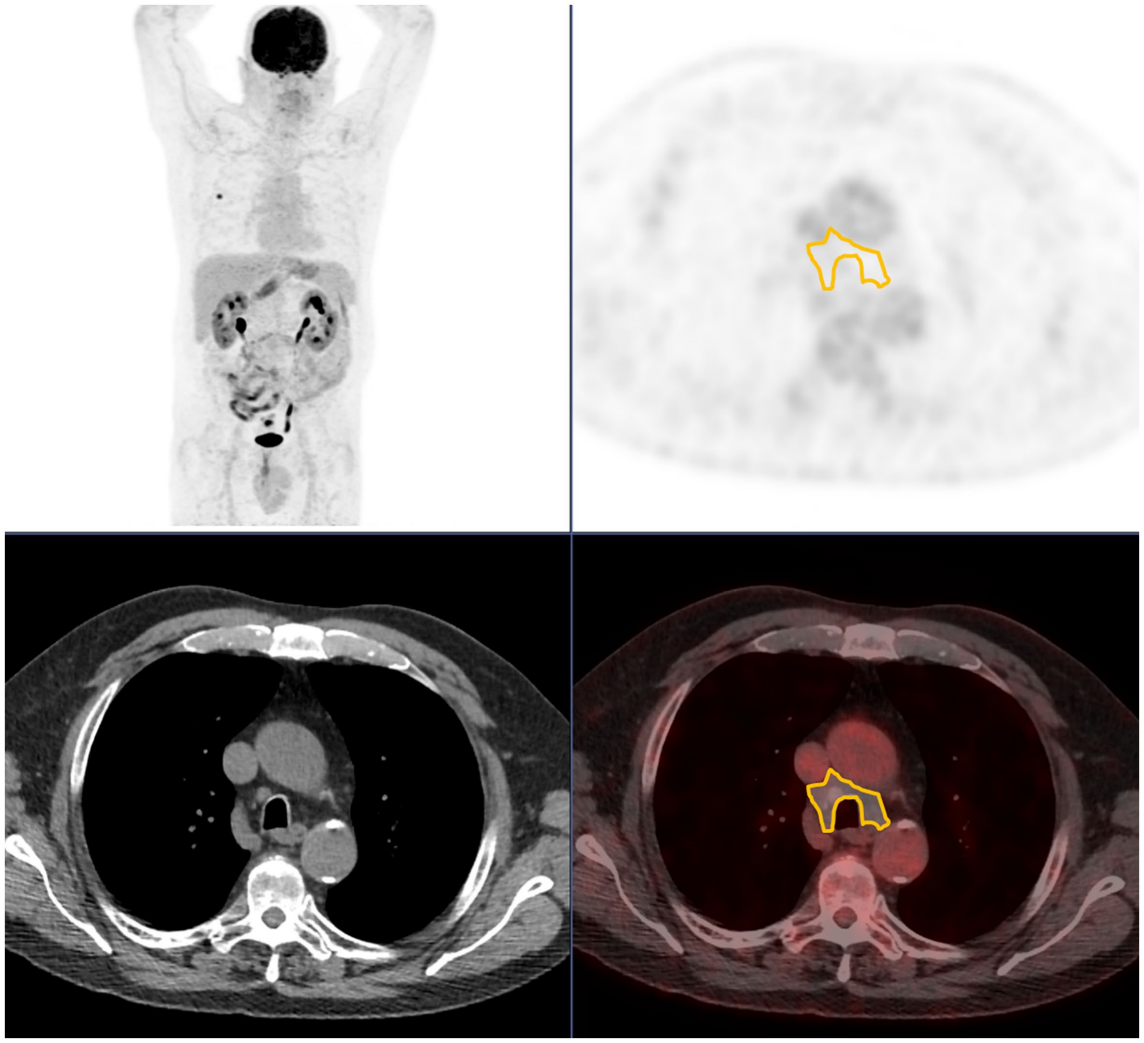
Region of interest used when no pathological lymph node uptake is seen, encompassing 4R and 4L station. From top to bottom and from left to right: maximum intensity projection; axial PET; axial CT; axial fused PET/CT.

SUVmax was measured using a manually drawn encompassing region of interest (ROI). For N_SUVmax, if no LNI was identified, an ROI encompassing right and left inferior paratracheal groups (4R-4L) was drawn (see Figure 1). Discrepancies between the measurements were resolved by consensus.

The 8th TNM classification was used (1), and pathological results were updated if an earlier version was used.

### 2.5. Statistical analysis

Quantitative data were described as mean and standard deviation (SD) and qualitative data as number and percentage. Comparison between training and validation set was performed using Student’s *t*-test, two proportion *Z*-test, or Fisher’s test when appropriate. In the training set, logistic regression was used to derive odds ratios (ORs) and 95% confidence intervals (95%CIs) on both univariate and multivariate analyses. The best multivariate model to predict pN0 status was selected based on Akaike’s Information Criterion. A receiver operating characteristic (ROC) curve was drawn to derive the area under the curve (AUC) and threshold. A threshold that favors specificity (least number of false positives, i.e., patients falsely predicted as pN0) while keeping sensitivity above 50% was chosen. False positives were reported, either when using the model or the visual interpretation alone and compared using a McNemar test.

In the validation set, an ROC curve using the identified optimal model was drawn to derive AUC. False positives, specificities, and positive predictive values were reported, either when using the model or the visual interpretation alone. The numbers of false positives were compared using a McNemar test based on a 2 × 2 contingency table (false positive vs. non-false positive, visual analysis vs. model). As SUV may be dependent on the machine used to acquire the PET/CT, the calculations were performed two times as follows: once without and once with a harmonization procedure. We used the ComBat algorithm (15) to perform this harmonization.

## 3. Results

### 3.1. Included patients

A total of 62 non-metastatic cT1 patients were identified in the training set. In total, 18 (29%) patients were excluded (10 benign findings, four lepidic adenocarcinomas, two neuroendocrine tumors, and two composite carcinomas with neuroendocrine component). In the end, 44 patients were included in the training set.

For the validation set, 118 cT1 cM0 patients with available PET/CT and pathological N status were identified, and eight patients with neuroendocrine tumors and two patients with missing data were excluded. A total of 108 patients were included.

Patients’ characteristics are presented in Table 1. The validation set included significantly older patients with more female patients (52.8% vs. 25.0%), higher adenocarcinoma frequency (78.7% vs. 61.4%), and less LNI both on clinical and pathological assessments.

**Table 1:** Patients’ characteristics.

	Training set	Validation set	Comparison
	(*n* = 44)	(*n* = 108)	
Age years (SD)	61.0 (8.4)	68.9 (9.2)	<0.001*
Sex number (%)			
Male	33 (75.0%)	57 (47.2%)	0.002*
Female	11 (25.0%)	51 (52.8%)	
Histological subtype number (%)			
Adenocarcinoma	27 (61.4%)	85 (78.7%)	
Epidermoid carcinoma	13 (29.5%)	22 (20.4%)	0.012*
Other	4 (9.1%)	1 (0.9%)	
Clinical T status number (%)			
cT1a	11 (25.0%)	20 (18.5%)	
cT1b	14 (31.8%)	52 (48.2%)	0.17
cT1c	19 (43.2%)	36 (33.3%)	
Primary tumor location number (%)			
Upper Right Lobe	7 (15.9%)	–	–
Centrally located	3 (6.8%)	–	–
PET/CT parameters			
cN0 number (%)	30 (68.2%)	100 (92.6%)	0.002*
T_SUVmax mean (SD)	7.6 (4.7)	6.9 (5.4)	0.444
N_SUVmax mean (SD)	3.1 (2.7)	–	–
Pathological N status number (%)			
pN0	27 (61.4%)	94 (87.0%)	
pN1	6 (13.6%)	8 (7.4%)	0.001*
pN2	11 (25.0%)	6 (5.6%)	
pN3	0 (0%)	0 (0%)	

SD, standard deviation. *p < 0.05.

### 3.2. Model development

In univariate analysis, only N_SUVmax and cN0 (visual analysis) were significant predictors of pN0 status (*p* < 0.001). N_SUVmax OR was 0.10 (the higher the N_SUVmax, the lower the probability of pN0), and cN0 OR was 84.55 (increased probability of pN0 if cN0). T_SUVmax and T status even not significant, had a value of *p* < 0.1. Age, primary tumor location, and sex were not predictive of pN0 status (Table 2). The best model derived from those parameters associated T_SUVmax, even not significant, and cN0 binary status. The derived equation giving the probability P of being pN0 was as follows:


$$P=1/\left[1+\exp\left(\begin{array}{l} 1,383842+0,168884\times \\ T\_SUVmax-4,602946\times cN0 \end{array}\right)\right]$$

**Table 2:** Prediction of pN0 status in cT1M0 patients: univariate and multivariate analyses.

	Univariate analysis	Multivariate analysis
		(Best Model)
Upper right lobe location	1.23 [0.24–6.34] *p* = 0.803	–
Peripheral location	3.47 [0.29–41.53] *p* = 0.309	–
Age	1.03 [0.96–1.11] *p* = 0.439	–
T (1a, 1b, 1c)	0.46 [0.20–1.06] *p* = 0.054	–
Sex (F)	1.96 [0.44–8.77] *p* = 0.363	–
T_SUVmax	0.88 [0.77–1.01] *p* = 0.065	0.85 [0.67–1.07] *p* = 0.139
N_SUVmax	0.10 [0.02–0.62] *p* < 0.001	–
cN0	84.55 [8.56–834.61] *p* < 0.001	99.78 [8.49–1172.90] *p* < 0.001

**Figure 2:**
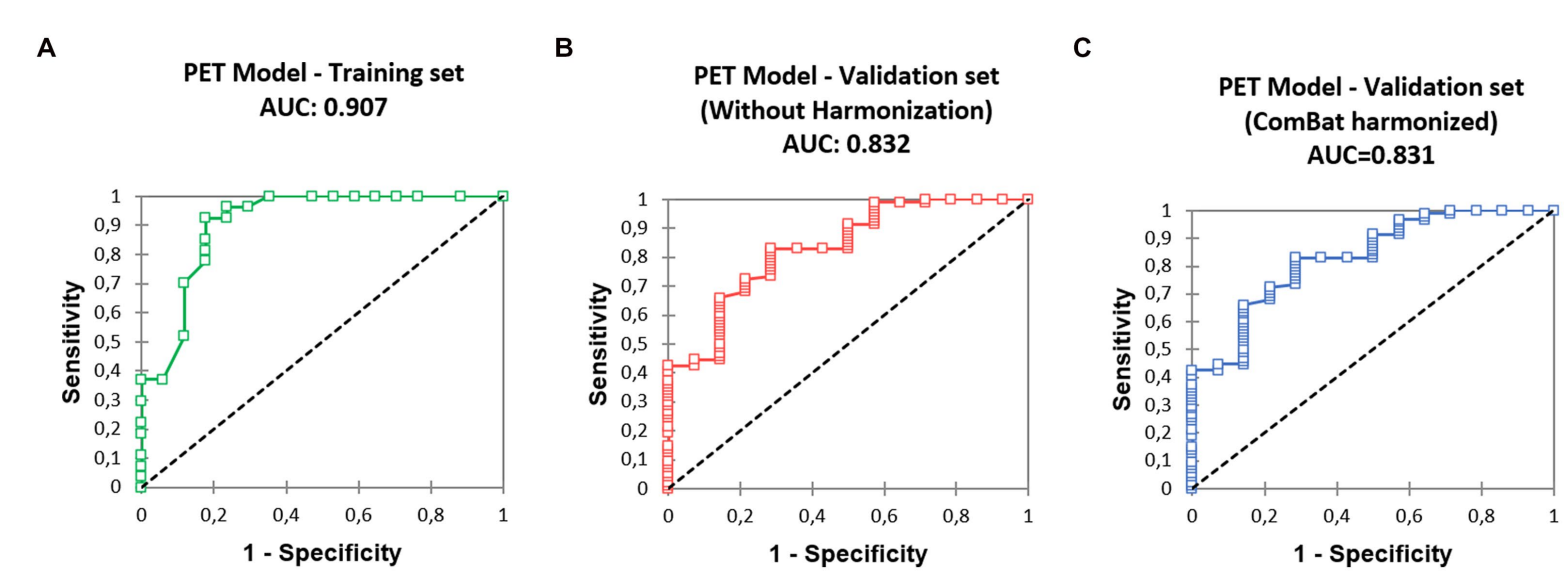
Receiver operating characteristic (ROC) curves derived from the predictive model, **(A)** training set, **(B)** validation set, without SUV harmonization, **(C)** validation set after SUV harmonization.

The corresponding ROC curve (Figure 2) had an AUC of 0.907 [0.809–1.000]. The optimal threshold to maximize the specificity of the model was 90%, with N0 status being predicted when *P* ≥ 90%. Derived sensitivity and specificity were, respectively, 0.519 [0.340–0.692] and 0.882 [0.642–0.977].

The whole training population (Table 1, *n* = 44) comprised six pN1 patients (13.6%) and 11 pN2 patients (25.0%). The use of visual analysis alone predicted 30 N0 patients, resulting in four false positives: four pN1 (13.3%) and 0 pN2 (0.0%). When applying our model, 16 patients were predicted as N0, resulting in two pN1 (12.5%) false positives. The specificity of the visual analysis approach was 0.765, and the specificity of the model was 0.882 (*p* = 0.480, Table 3). A total of five patients (11.3%) were upstaged from cT1 to pT2 based on measurement uncertainty.

**Table 3:** Specificity and positive predictive value of visual analysis and model-based prediction.

	Training cohort			Validation cohort		
	Visual analysis	Model-based prediction	Comparison (false positive number)	Visual analysis	Model-based prediction	Comparison (false positive number)
Specificity	76.5%	88.2%	*p* = 0.480	65.4%	92.3% (92.3%)	*p* = 0.023*
False pN0 prediction rate						
Positive predictive value	86.7%	87.5%		91.0%	96.4% (95.7%)	(*p* = 0.023*)
% of predicted N0 that are pN0						

Data using harmonized SUV are presented in parenthesis; *p < 0.05.

### 3.3. Model validation

#### 3.3.1. Validation using native, unharmonized SUV data

The ROC curve (Figure 2) calculated on the validation cohort showed an AUC of 0.832 [0.721, 0.944]. The whole population (*n* = 108) comprised eight pN1 (7.4%) and six pN2 (5.6%). Using visual analysis only, 100 patients were predicted to be N0, resulting in nine false positives: four pN1 (4.0%) and five pN2 (5.0%). When using the predictive model, 56 patients were predicted as N0, with two false positive patients: one pN1 (1.8%) and one pN2 (1.8%). Specificities were 0.654 (visual analysis) and 0.923 (model), significantly different (*p* = 0.023, Table 3). In total, 18 (16.7%) patients classified as cT1 were not pT1 after surgery: 16 patients were pT2 (size difference < 5 mm between estimation and pathological measure); one was pT3 (unseen separate nodule adjacent to the main primary); and one was pT4 (due to adjacent organs invasion).

#### 3.3.2. Validation using harmonized SUV data

The ComBat algorithm was used to determine corrected SUV (corSUV) corresponding to the SUV that would have been found if the patients in the validation cohort (Biograph mCT PET/CT—Siemens) had undergone the examination under the machine used in the training cohort (Discovery ST system—General Electrics).

The ROC curve (Figure 2) calculated on the validation cohort showed an AUC of 0.831 [0.720; 0.942]. When using the predictive model with corSUV, 46 patients instead of 56 were predicted as N0. All those 46 patients were considered as N0 using unharmonized SUV. The remaining discrepant 10 patients were all pN0. The number of false positives was thus unchanged.

## 4. Discussion

Occult lymph node involvement on 18F-FDG PET/CT is a central problem in the management of lung cancer, with an estimated average occult N2 rate for stage I tumors of 6.1% (16).

### 4.1. Positron emission tomography model

Successive studies have attempted to determine new parameters to decrease this proportion, mainly based on size, location, and uptake of the primary tumor. Our model retained only two parameters among these factors as follows: the visual interpretation of the lymph node status by the nuclear medicine physician (cN0 vs. cN1, cN2, or cN3) and the SUVmax of the primary tumor. In this study, we propose a simple algorithm to detect a subpopulation with a very low risk of lymph node involvement in two steps as follows:

1. Check the eligibility of the patient: measure the primary tumor (< 3 cm, corresponding to a cT1) and ensure the absence of metastasis.
2. Use the model: the model equation (eq. 1.), although complex, can be simplified and decomposed by noting that the condition *P* ≥ 0.9 can only be met if a patient fulfills two conditions: being cN0 and having a T_SUVmax of less than 6.05 without harmonization procedure.

Using this algorithm, the proportion of occult LNI was significantly reduced: 3.6% (N1 or N2) and 1.8% (N2 only). The false positive rate, even lower when using the algorithm, was not significantly different on the training dataset in comparison to visual analysis, presumably due to the sample size of the training set. However, it reached significance in the validation set.

We considered only the clinical T-stage, which is the only one accessible preoperatively. This measure is, however, a source of uncertainty, as shown by the proportion of upstaging after surgery encountered in our two cohorts (between 10 and 20%). The majority of upstaging concerned cT1 tumors with a size close to 3 cm, the limit of the T2 stage.

The T_SUVmax threshold of 6.05 is not directly comparable to the one reported in other studies: we did not seek an optimal threshold but a threshold minimizing the number of occult lymph nodes. However, it is interesting to note that this threshold remains in the same range as those previously reported between 4 (13) and 7.5 (12). The study by Vansteenkiste et al. (17), although not directly concerning occult LNI, reported a better 2-year survival when the primary tumor had SUV lesser than 7 and tumor size lower than 3 cm. Like all SUV-based indices, the concern of inter-machine generalization is raised. However, our model showed similar good performances, with and without harmonization showing some robustness to the change of reconstruction protocol and machine. This problem could be more present with the new digital PET/CT, given their better technical characteristics in terms of resolution and sensitivity. Harmonization will, then, probably be systematically required.

### 4.2. Potential clinical implications

From a theoretical point of view, the model presented herein would allow a better selection of patients who are candidates for minimally invasive staging or more conservative intra-operative lymph node dissection with several practical implications: optimizing resources and reducing costs; avoiding complications and delay due to unnecessary procedures. Obviously, these results need to be confirmed in a prospective clinical cohort of patients.

### 4.3. Limitations

Several limitations need to be discussed. Apart from the limitations inherent in the retrospective nature of the study, our training cohort presents some notable differences from our validation cohort, mainly related to the period of patient recruitment (2004 to 2009 vs. 2018 to 2021). In particular, the proportion of women is increased in the validation cohort, consistent with the increased prevalence of lung cancer in this population; this increase probably explains the increased proportion of lung adenocarcinomas, which are more frequent in the female population. The age of the validation population was significantly higher with a delta of 7 years, probably due to a center effect, as was the proportion of cN0. Despite these differences, the good performance of the model is reassuring.

Several factors were not taken into account for the measure of N_SUV, particularly the background uptake level and the partial volume effect. Indeed, we tried to use a method of measurement as simple as possible to be applied in clinical routine. In addition for simplicity, we estimated lymph node uptake only by measuring the lymph node with the highest uptake not by measuring all the most frequently affected mediastinal lymph nodes.

## 5. Conclusion

A model associating the SUVmax of the primary tumor with the lymph node visual interpretation allows to reduce the number of occult adenopathy in early-stage NSCLC.

## Data availability statement

The raw data supporting the conclusions of this article will be made available by the authors, without undue reservation.

## Ethics statement

The studies involving human participants were reviewed and approved by Institutional Ethics Committee Fondazione Policlinico Universitario A. Gemelli IRCCS —Università Cattolica del Sacro Cuore (registration number: 0015316–03/05/2022). Written informed consent for participation was not required for this study in accordance with the national legislation and the institutional requirements.

## Author Contributions

DM and AI: conceptualization and validation. DM: methodology, software, formal analysis, and writing – original draft preparation. DM, MC, and FL: data curation. DM, MC, P-EF, M-PC, SA, LB, FL, and AI: writing – review and editing. All authors have read and agreed to the published version of the manuscript.

## Conflict of interest

The authors declare that the research was conducted in the absence of any commercial or financial relationships that could be construed as a potential conflict of interest.

## Publisher’s note

All claims expressed in this article are solely those of the authors and do not necessarily represent those of their affiliated organizations, or those of the publisher, the editors and the reviewers. Any product that may be evaluated in this article, or claim that may be made by its manufacturer, is not guaranteed or endorsed by the publisher.
